# Quantification of Nasolacrimal Duct Obstruction Length and Its Relationship with Surgical Outcomes

**DOI:** 10.3390/jcm15020853

**Published:** 2026-01-20

**Authors:** Yoshiki Ueta, Yuji Watanabe, Nobuya Tanaka, Anzu Tanaka

**Affiliations:** Department of Ophthalmology, Shinseikai Toyama Hospital, 89-10 Shimowaka, Imizu 939-0243, Toyama, Japan

**Keywords:** nasolacrimal duct obstruction, calibrated dacryoendoscope, endoluminal lacrimal duct recanalization, nasolacrimal duct obstruction length, cotton thread test

## Abstract

**Background/Objectives:** Although the outcomes of endoluminal lacrimal duct recanalization (ELDR) for nasolacrimal duct (NLD) obstruction are associated with the obstruction length, NLD obstruction has not been quantified. In this study, we aimed to quantify the obstruction length using a calibrated dacryoendoscope and investigate its relationship with surgical outcomes following endoscopic recanalization of the lacrimal passage. **Methods:** We retrospectively analyzed the eyes of patients who underwent ELDR using a calibrated dacryoendoscope at our institution between January 2023 and February 2025. Patients with dacryocystitis detected during preoperative irrigation testing were excluded. A lacrimal tube was placed for 2 months after recanalization, and we used the calibrated dacryoendoscope to measure the obstruction length. The 3-month postoperative outcomes were determined using irrigation testing, subjective symptoms, and cotton thread testing. **Results:** A total of 31 eyes of 26 patients (6 eyes of 5 males; 25 eyes of 21 females; mean age, 68.8 ± 10.5 years) were included in this study. The mean obstruction length was 10.8 mm (range: 1–30 mm). The nasolacrimal duct was patent, showed reflux, and was obstructed in 12, 16, and 3 eyes, respectively, at 3 months. The symptoms resolved, improved, and remained unchanged in 18, 11, and 2 eyes, respectively. Trend analysis revealed a significant relationship between the obstruction length and irrigation outcomes. However, no significant association was observed with the symptoms. The obstruction length was significantly correlated with the 3-month postoperative cotton thread test results. **Conclusions:** The NLD obstruction length was associated with the 3-month postoperative irrigation test results, and longer obstruction was associated with poorer postoperative outcomes.

## 1. Introduction

Epiphora is one of the main symptoms of patients visiting ophthalmology clinics and is most commonly caused by lacrimal drainage obstruction, which does not improve with conservative treatment and requires surgical intervention. Lacrimal drainage obstruction can be broadly classified as pre-saccular or post-saccular, for which the main treatments are endoluminal lacrimal duct recanalization (ELDR) and dacryocystorhinostomy (DCR), respectively. The reported success rate of DCR for acquired nasolacrimal duct obstruction is high, ranging from 93% to 100% [1,2,3,4]. Recently, ELDR has also been applied to nasolacrimal duct (NLD) obstruction, and favorable outcomes have been reported [5,6,7,8,9,10,11,12,13]. ELDR is generally less invasive than DCR and can be completed within a shorter duration. The poor prognostic factors of intraluminal reconstruction surgery for NLD obstruction include coexisting dacryocystitis [5,7,10], long-segment NLD obstruction [8,11], and prolonged duration of epiphora [7].

NLD obstruction has not been quantified, and assessments have relied on qualitative classifications such as complete versus partial obstruction [11] and proximal versus distal obstruction [8].

We aimed to address this limitation by developing a calibrated dacryoendoscope capable of quantifying obstruction length. We report the quantitative measurement of this length and its relationship with the outcomes of endoscopic recanalization. To the best of our knowledge, this is the first study to quantitatively measure the length of NLD obstruction using a calibrated dacryoendoscope, which may provide new insights into its pathophysiology and help improve surgical outcomes.

## 2. Patients and Methods

### 2.1. Ethics

This study was conducted in accordance with the tenets of the Declaration of Helsinki and approved by the Institutional Ethical Review Board of Shinseikai Toyama Hospital (approval number: 2024-015). We used an opt-out consent process, which involved providing the participants with full written information about the study. They were included unless they expressed their decision to be excluded. The full written information was approved by the Institutional Review Board of Shinseikai Toyama Hospital, which also waived the requirement for obtaining informed consent.

### 2.2. Patients

We retrospectively reviewed the cases of NLD obstruction treated via ELDR using a calibrated dacryoendoscope at our institution between January 2023 and December 2024. The diagnosis of NLD obstruction was based on complete blockage revealed by preoperative irrigation testing and confirmed intraoperatively via dacryoendoscopy. The exclusion criteria were chronic dacryocystitis (defined as purulent reflux on irrigation), punctal obstruction, previous lacrimal surgery, congenital NLD obstruction, traumatic obstruction, and a follow-up duration of less than 3 months. Cases in which the obstruction could not be penetrated intraoperatively were also excluded, as its length could not be measured. Although some cases had concomitant common canalicular stenosis or obstruction, all included eyes had primary nasolacrimal duct obstruction confirmed intraoperatively. The measured obstruction length represented the length of obstruction within the nasolacrimal duct, not the canalicular system.

### 2.3. Instruments

**Figure 1:**
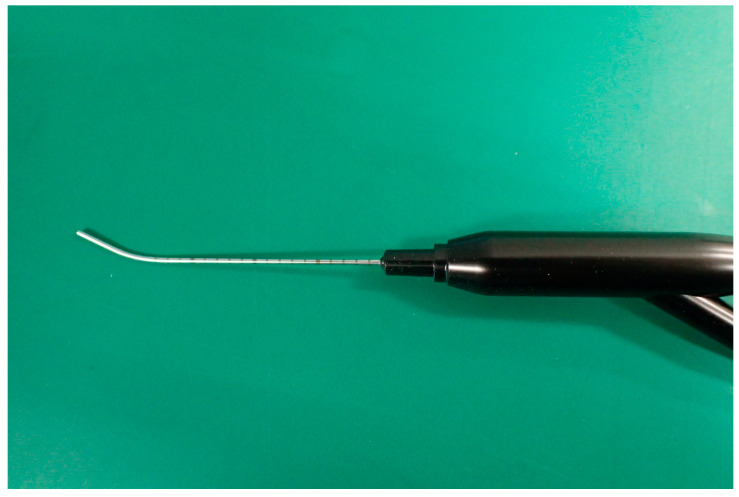
Calibrated dacryoendoscope. A newly developed bent-type dacryoendoscope with engraved calibration marks. Scale markings were placed at 2 mm intervals, with bold markings at 10 mm intervals. These facilitated the quantitative measurement of the obstruction length in the nasolacrimal duct.

The calibrated dacryoendoscope (developed in collaboration with FiberTech Co., Tokyo, Japan) had a length of 50 mm, diameter of 0.9 mm, resolution of 10,000 pixels, and focal distance of 1.5–7 mm. It was a bent-type scope with 2 mm interval markings and bold markings at 10 mm intervals engraved using laser to prevent toxicity and ensure durability (Figure 1).

### 2.4. Surgical Procedure

All surgeries were performed by a single surgeon (Y.U.) under local anesthesia, which was achieved via a trochlear nerve block on the operated side and infiltration around the nasolacrimal duct using 1% lidocaine with epinephrine. A mixture of 4% lidocaine and 1% epinephrine was applied to the nasal cavity using a Jackson-type laryngeal spray (Taiyu Medical Instruments Co., Ltd., Osaka, Japan), and a cotton swab soaked in 4% lidocaine was placed in the inferior nasal meatus.

The punctum was dilated after anesthesia, and the calibrated dacryoendoscope was inserted via the upper punctum. The upper end of the obstruction was identified, and its distance from the punctum was determined using the scale on the endoscope. The obstructed site was perforated via a direct endoscopic procedure. The lower end of the obstruction was determined after perforation of the blocked segment. The obstruction length was defined as the distance between the upper and lower ends (the distance from the punctum to the proximal end of the obstruction was subtracted from that to the distal end). Canalicular lesions were not included in the measurement. Video S1 shows the measurement of the distance to the upper end of the obstruction using the calibrated dacryoendoscope. A Nunchaku-style stent tube (N-ST^®^, Carl Zeiss Meditec AG, Jena, Germany) was subsequently inserted via direct silicone intubation (DSI) or sheath-guided intubation when DSI was not feasible. The tubes were left in place for 2 months.

The patients received topical cefmenoxime (four times daily for 2 weeks) and 0.1% fluorometholone (four times daily for 3 months) postoperatively.

### 2.5. Examinations

The patients were evaluated at 2 weeks, 2 months, and 3 months postoperatively, and irrigation testing was performed at each visit. The outcomes were classified as good (free passage of saline into the nasal cavity without reflux), fair (passage of saline into the nasal cavity with partial reflux through the punctum), or obstructed (no passage of saline with complete reflux). The subjective symptoms were classified as resolved, improved, or unchanged. Tear quantity was measured using the cotton thread test (CT-T, Zone-Quick^®^, Showa Yakuhin Kako, Tokyo, Japan) preoperatively and at 3 months after surgery.

### 2.6. Outcome Measures

For statistical analyses, surgical success was defined as patency on irrigation, which encompassed good or fair outcomes. Both types of outcomes indicated functional tear drainage despite the presence of partial reflux in the fair group. This definition was adopted to reflect clinically meaningful improvement in lacrimal drainage. The primary outcome was the relationship between obstruction length and irrigation results at 3 months. The secondary outcomes included the overall success rate, symptom improvement at 3 months, changes in CT-T before and 3 months after surgery, and correlations among obstruction length, symptoms, irrigation results, and CT-T values at 3 months.

### 2.7. Statistical Analysis

Data are summarized as the mean ± SD. The Jonckheere–Terpstra test was used to determine the trends in the relationships between obstruction length, irrigation results, symptoms, and CT-T values at 3 months after surgery. The Wilcoxon signed-rank test was used to compare the preoperative and postoperative CT-T values, and Spearman’s rank correlation coefficient was used to assess the correlation between obstruction length and CT-T values.

## 3. Results

The data of 31 eyes from 26 patients (6 eyes from 5 men and 25 eyes from 21 women) with a mean age of 68.8 ± 10.2 years were included in the analysis. The affected eye was right for 14 and left for 17 cases. Combined common canalicular stenosis and common canalicular obstruction were observed in two and four eyes, respectively (Table 1). The mean nasolacrimal obstruction length determined via calibrated dacryoendoscopy was 11.0 ± 7.7 mm (range, 1–30 mm).

**Figure 2:**
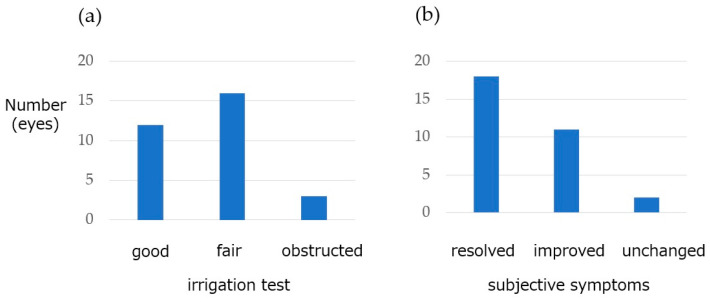
Overall surgical outcomes at 3 months. (**a**) The irrigation test results were classified as good (patent without reflux), fair (patent with reflux), or obstructed. Good and fair outcomes accounted for 90.3%. (**b**) The subjective symptom outcomes were classified as resolved, improved, or unchanged. The symptoms of 93.5% of the cases improved.

**Figure 3:**
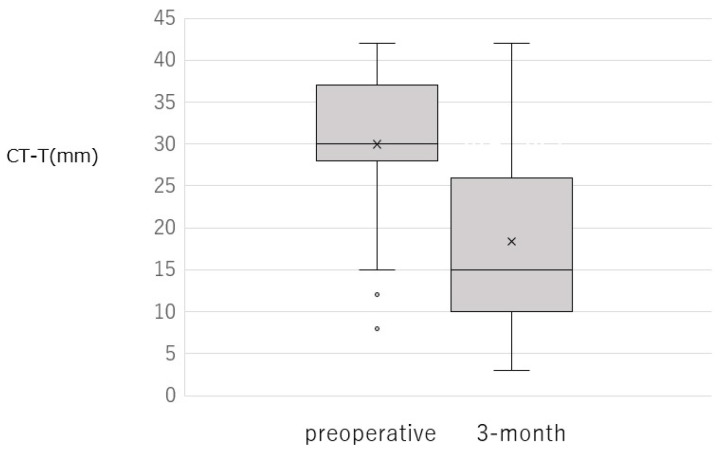
Comparison of cotton thread test results preoperation and at 3 months postoperation, showing significant reduction in tear retention (*p* = 0.0000187).

Irrigation testing at 3 months after surgery revealed patent passage without reflux, passage with reflux, and obstruction in 12 (38.7%), 16 (51.6%), and 3 (9.7%) eyes, respectively (Figure 2). The overall success rate was 90.3% (28 of 31 eyes) based on successful passage with or without reflux. The subjective symptoms improved for most patients: they completely resolved in 18 eyes (58.1%), improved in 11 (35.5%), and remained unchanged in 2 (6.5%). The cotton thread test (CT-T) also demonstrated significant improvement, with the mean wetting length decreasing from 30.0 ± 8.9 mm preoperatively to 18.4 ± 10.9 mm at 3 months postoperatively (*p* = 0.0000187) (Figure 3).

**Figure 4:**
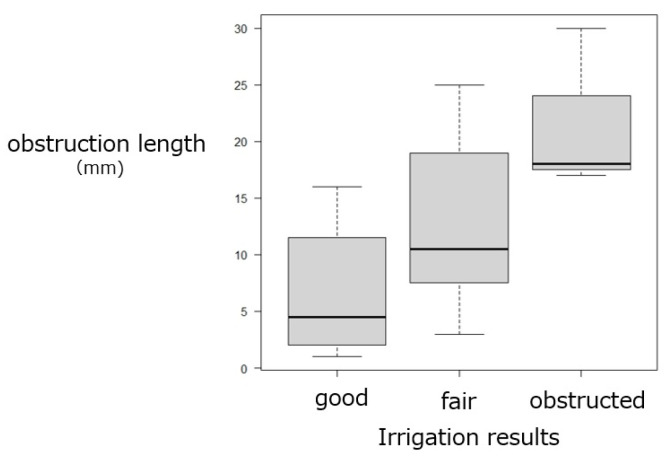
Relationship between irrigation results and obstruction length. Trend analysis revealed shorter obstruction lengths in cases with patent irrigation and longer lengths in cases with obstruction (*p* = 0.00104).

**Figure 5:**
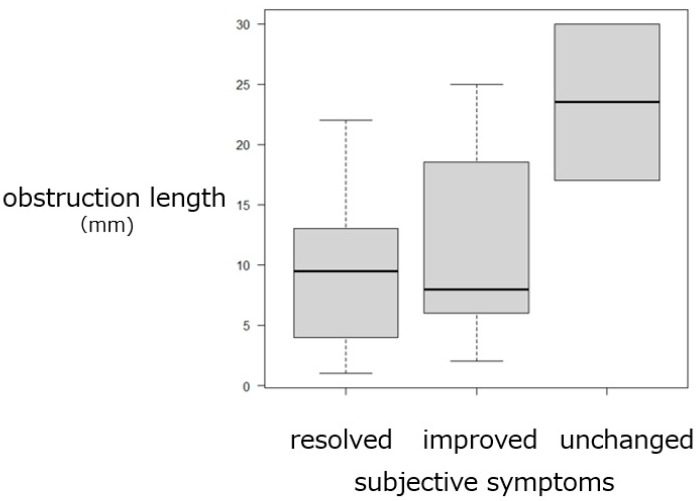
Relationship between obstruction length and subjective symptoms being resolved, improved, or unchanged postoperatively. The obstruction length was not significantly associated with the postoperative symptoms (*p* = 0.136).

**Figure 6:**
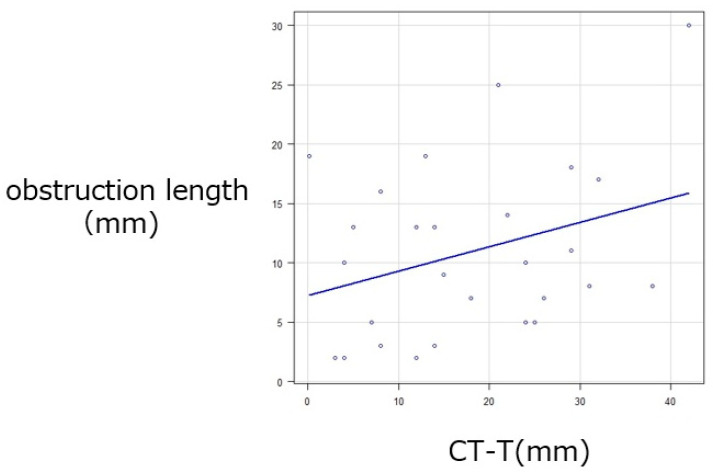
Correlation between obstruction length and cotton thread test results at 3 months, showing no significant correlation (*p* = 0.209). The blue line represents the linear regression line.

**Figure 7:**
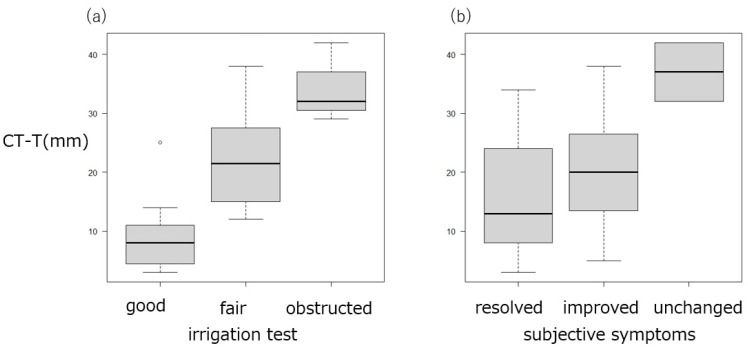
Relationship between postoperative cotton thread test results, irrigation outcomes, and subjective symptoms. (**a**) The CT-T values for the eyes with better irrigation outcomes were significantly lower at 3 months. (**b**) The CT-T values for the eyes with resolved or improved symptoms were significantly lower than those for the eyes with unchanged symptoms at 3 months. Both sets of outcomes were significantly associated with postoperative CT-T results (*p* = 0.00000625 and 0.0206).

Trend analysis revealed significant associations between obstruction length and irrigation outcomes. Shorter obstructions were more frequently observed in eyes with patent passage, whereas longer obstructions were characteristic of those with persistent blockage (*p* = 0.00104) (Figure 4). In contrast, the obstruction length was not associated with postoperative symptoms (*p* = 0.136) (Figure 5) and was not significantly correlated with the postoperative CT-T values at 3 months (*p* = 0.209) (Figure 6). In contrast, both irrigation results and subjective symptom improvement were significantly associated with postoperative CT-T outcomes (*p* = 0.00000625 and 0.0206, respectively) (Figure 7). These associations indicate that the patients with more favorable irrigation results and better symptomatic relief had lower CT-T values.

## 4. Discussion

In this study, we developed a calibrated dacryoendoscope for quantifying NLD obstruction length and evaluated its relationship with surgical outcomes following lacrimal passage recanalization. We used laser engraving to create the scale to prevent potential effects of paint on biological tissues. The markings were engraved without shaving the metal pipe to preserve their strength and durability. Our findings demonstrated that obstruction length significantly influenced the irrigation test results at 3 months after surgery, and longer obstructions were associated with poorer outcomes.

The relationship between obstruction length and prognosis has been suggested in reports of previous studies [8,11]. However, these have relied primarily on qualitative classifications such as complete versus partial obstruction or proximal versus distal involvement. This study provides novel evidence supporting the use of obstruction length as a prognostic marker. Shorter obstructions may reflect localized fibrotic changes within the duct, which are associated with easier traversal and stabilization after recanalization. In contrast, longer obstructions are more likely to indicate extensive fibrosis and structural alteration of the ductal wall and are associated with an increased risk of restenosis or surgical failure.

The success rate in this study was 90.3%, which is higher than the previously reported rates of 71.4–86.2% [5,6,7,8,9,10,11,12,13]. Several factors may explain this discrepancy. First, cases with chronic dacryocystitis were excluded. This is because dacryocystitis is associated with impaired surgical prognosis due to inflammatory changes and infection of the sac mucosa. Second, our analysis included only cases in which intraoperative recanalization was technically achievable. Consequently, more severe cases in which perforation could not be achieved intraoperatively were excluded. Third, the postoperative follow-up duration was limited to 3 months, and longer-term recurrence may reduce the observed success rate. Therefore, the short-term results are promising, but their durability should be confirmed through longer follow-up.

No significant association was observed between obstruction length and postoperative subjective symptoms. This discrepancy may reflect the relatively high overall success rate, as even partial tear passage can alleviate symptomatic epiphora. Moreover, subjective symptoms are inherently variable and influenced by patient perception. Therefore, they may not always correspond strictly to anatomical findings.

Tear meniscus height is commonly used to assess tear volume. However, its quantification requires anterior segment optical coherence tomography (AS-OCT), which is not available at all facilities. Therefore, we used the CT-T to evaluate tear volume in the present study. This measures the amount of tear fluid present at the time of testing, whereas the Schirmer test, which uses a similar approach, evaluates tear secretion. Several reports have described the use of the CT-T for dry eye disease evaluation [14,15,16]; its utility is considered to be lower than that of the Schirmer test in this context, given the critical role of tear secretion in dry eyes. However, to our knowledge, the CT-T has not been applied to epiphora, in which tear retention is more important than tear secretion. Therefore, we considered this test to be useful for evaluating this condition. The significant associations of the CT-T results with the irrigation outcomes and subjective symptoms at 3 months postoperatively highlight the value of the CT-T for evaluating tear volume in patients with epiphora. However, no significant correlation was observed between the obstruction length and the results of this test. This finding may be explained in the same way as the subjective symptoms. Tear retention may remain relatively low if the nasolacrimal duct is narrowed but a degree of patency is maintained.

This study has some limitations. First, it was retrospective and involved a relatively small number of cases, which may limit the generalizability of the findings. Second, the follow-up duration was limited to 3 months, and longer-term outcomes, including delayed reobstruction, could not be assessed. Third, errors may have occurred in identifying the lower margin of the obstruction; precise localization is more challenging for this margin than it is for the upper margin. Although all surgeries were performed by a single surgeon and via the upper punctum, the measurements may have varied depending on whether the superior or inferior punctum was used for insertion. The irrigation results and subjective symptoms were categorized into three classifications, but they were not quantified in detail. Future studies incorporating more refined grading systems may provide better insights into these relationships.

The development of our calibrated dacryoendoscope represents a significant advance in the objective evaluation of NLD obstruction. This tool will enable the measurement of obstruction length, will facilitate more accurate prognostication, and may contribute to improved surgical planning and patient counseling. Future prospective studies with larger sample sizes and longer follow-up are needed to confirm the findings of this study and establish obstruction length as a standard prognostic parameter for the management of NLD obstruction.

## 5. Conclusions

We developed a calibrated dacryoendoscope that enabled quantitative assessment of NLD obstruction length, which was significantly associated with the irrigation outcomes at 3 months; longer obstructions indicated poorer prognoses. The novel quantification approach used in this study will provide valuable insights into the pathophysiology and treatment of NLD obstruction.

## Figures and Tables

**Table 1:** Characteristics of the patients.

Number	26 Patients (31 Nasolacrimal Ducts): 5 Males, 6 Eyes; 21 Females, 25 Eyes
Age	68.8 ± 10.2 years old
Laterality (right-to-left)	14:17
Concomitant common canalicular stenosis	2 eyes
Concomitant common canalicular obstruction	4 eyes
Length of obstruction	11.0 ± 7.7 mm (1–30 mm)

Summary of the demographic and clinical characteristics of the patients who underwent dacryoendoscopic recanalization using a calibrated dacryoendoscope.

## Data Availability

All data analyzed in this study are included in this article. Further inquiries can be directed to the corresponding author.

## Supplementary Materials

The following supporting information can be downloaded at: https://www.mdpi.com/article/10.3390/jcm15020853/s1. Video S1: The measurement of the distance to the upper end of the obstruction using the calibrated dacryoendoscope.

## Abbreviations

The following abbreviations are used in this manuscript:

DCR	dacryocystorhinostomy
CT-T	cotton thread test
DSI	direct silicone intubation
ELDR	endoluminal lacrimal duct recanalization
NLD	nasolacrimal duct
